# Efficacy of full-fat milk and diluted lemon juice in reducing infra-cardiac activity of ^99m^Tc sestamibi during myocardial perfusion imaging

**DOI:** 10.5830/CVJA-2015-033

**Published:** 2015

**Authors:** Khushica Purbhoo, Mboyo Di Tamba, Willy Vangu

**Affiliations:** Department of Nuclear Medicine and Molecular Imaging, Chris Hani Baragwanath Academic Hospital and Charlotte Maxeke Johannesburg Academic Hospital, University of the Witwatersrand, Johannesburg, South Africa; Department of Nuclear Medicine and Molecular Imaging, Chris Hani Baragwanath Academic Hospital and Charlotte Maxeke Johannesburg Academic Hospital, University of the Witwatersrand, Johannesburg, South Africa; Department of Nuclear Medicine and Molecular Imaging, Chris Hani Baragwanath Academic Hospital and Charlotte Maxeke Johannesburg Academic Hospital, University of the Witwatersrand, Johannesburg, South Africa

**Keywords:** myocardial perfusion imaging, full-fat milk, lemon juice, infra-cardiac activity, sestamibi, ^99m^Tc

## Abstract

**Background:**

When using ^99m^Tc sestamibi for myocardial perfusion imaging, increased splanchnic activity creates a problem in the visual and quantitative interpretation of the inferior and infero-septal walls of the left ventricle. We sought to determine whether the administration of diluted lemon juice or full-fat milk would be effective in reducing interfering infra-cardiac activity and therefore result in an improvement in image quality. We compared the administration of full-fat milk and diluted lemon juice to a control group that had no intervention.

**Methods:**

The study was carried out prospectively. All patients referred to our institution for myocardial perfusion imaging from November 2009 to May 2012 were invited to be enrolled in the study. A total of 630 patients were randomised into three groups. Group 0 (G0), 246 patients, were given diluted lemon juice, group 1 (G1), 313 patients, were given full-fat milk, and group 2 (G2), 71 patients, had no intervention (control group). A routine two-day protocol was used and the patients were given the same intervention on both days. Raw data of both the stress and rest images were visually assessed for the presence of infra-cardiac activity, and quantitative grading of the relative intensity of myocardial activity to infra-cardiac activity was determined. The physicians were blinded to the intervention received and the data were reviewed simultaneously.

**Results:**

The overall incidence of interfering infra-cardiac activity at stress was 84.1, 84.5 and 96.6% in G0, G1 and G2, respectively (*p* = 0.005). At rest it was 91.7, 90.1 and 100% in G0, G1 and G2, respectively (*p* = 0.0063). The visual and quantitative results favoured both milk and lemon juice in reducing the amount of interfering infra-cardiac activity versus no intervention.

**Conclusion:**

The administration of milk or lemon juice resulted in a significant decrease in the intensity of infra-cardiac activity compared to the control group. This reduction in intensity was even more significant in the milk group for patients assessed during rest myocardial perfusion imaging.

## Background

Coronary artery disease is one of the leading causes of death throughout the world. In most African countries, cardiovascular disease (CVD) is now the second commonest cause of death after infectious disease, accounting for 10% of total deaths, and it is estimated that this burden will double from 1990 to 2020.^1^,^2^ It also presents an enormous burden due to morbidity and health care expenses.

Myocardial perfusion imaging (MPI) is a valuable tool in the management of patients with CVD and is currently used in Africa.^3^ The use of single-photon emission computed tomography (SPECT) myocardial perfusion imaging, with technetium 99m-labelled radiopharmaceuticals [^99m^Tc sestamibi (methoxy isobutyl isonitrile) and ^99m^Tc tetrofosmin] in conjunction with either exercise or pharmacological stress is an established tool for both the diagnosis and prognostication of patients with ischaemic heart disease.^4^

The basis of the non-invasive approach is that physiological changes in regional myocardial blood flow or systolic contraction of the myocardium caused by stress may be more predictive of outcome than a knowledge of coronary anatomy alone. Patients with normal perfusion on ^99m^Tc SPECT MPI had an excellent prognosis, whereas patients with abnormal scans had an increased rate of cardiac death and non-fatal infarction during follow up.^5^

For perfusion imaging with SPECT, thallium 201 (^201^Tl) and ^99m^Tc-labelled radiopharmaceuticals are commonly used. The major metabolic pathway for clearance of sestamibi is the hepatobiliary system, therefore infra-cardiac activity from the liver and bowel may impact on the interpretation of the inferior wall after reconstruction. The presence of infracardiac activity leads to artifacts, reducing the desired targetto- background ratio, which creates difficulty in both visual and quantitative interpretation of myocardial perfusion.^5^ Activity may also be present in the stomach due to reflux of tracer into the gastric lumen from the duodenum, or because of uptake of free pertechnetate by the gastric mucosa. Infra-cardiac activity is less common with exercise and is more common with pharmacological stress and/or in rest studies.^6^

Several different protocols, including a fatty meal, drinking milk, milk and water, lemon juice, milkshake, carbonated drinks, iodinated oral contrast, intravenous injection of cholecystokinin, and the administration of metoclopramide or erythromycin have been described as a means to reduce the artifacts arising from abdominal activity.^7^-^16^ Depending on the protocol used, the mechanisms for these interventions include one or more of the following: expansion of the stomach by the volume effect, displacing it caudally, thereby increasing the distance between the heart and the bowel; increased gastric emptying; stimulation of liver clearance and peristaltic movement; and acceleration of bile secretion and gallbladder emptying.

The aim of this study was to evaluate the efficacy of lemon juice or milk administration compared to a control group, to decrease infra-cardiac activity and to assess any resultant effect on image interpretation of myocardial perfusion.

## Methods

This was a prospective study. All patients 18 years and older who were referred for MPI were invited to be enrolled in the study. Ethics approval was obtained from the University of the Witwatersrand’s Human Research Ethics Committee and written consent was obtained from all study participants.

The study commenced in November 2009 and ran until May 2012. We recruited 904 patients but data from 274 patients were excluded for various reasons [non-return for second day’s study, milk or lemon juice not followed in a patient for both the stress and rest study, and patients who fitted the exclusion criteria (Table 1)]. A total of 630 patients [304 female (48%) and 326 male (52%)] aged 19–84 years were eventually enrolled for data analysis.

**Table 1:** Inclusion and exclusion criteria

*Inclusion criteria*	Exclusion criteria
Patients older than 18 years of age	Lactose intolerance
Patients referred for ^99m^Tc sestamibi myocardial perfusion imaging	Patients who failed exercise stress testing and had a contra-indication to pharmacological stress testing, i.e. using vasodilators and dobutamine
	Unable to drink 250 ml of fluids secondary to medically essential fluid restriction
	Pregnant patients
	Previous cholecystectomy, liver or biliary system disease
	Peptic ulcer disease within the last six months
	History of diabetes mellitus
	Previous myocardial infarction within the last two months, unstable angina, severe primary valvular disease, left ventricular aneurysm, primary cardiomegaly, left ventricle hypertrophy or severe conduction disturbances

Patients were randomised into three groups. Group 0 (G0) drank diluted lemon juice, group 1 (G1) drank full-fat milk, and group 2 (G2) had no intervention (control group). Full-fat milk consisted of 250 ml milk. Diluted lemon juice consisted of 50 ml lemon juice and 200 ml water, with a total volume of 250 ml.

Following the injection of 740 MBq ^99m^Tc sestamibi during stress, patients in G0 received diluted lemon juice and patients in G1 received full-fat milk 20 minutes after the tracer injection, whereas patients in G2 received no intervention. After the rest injection of 740 MBq of ^99m^Tc sestamibi, patients in G0 received diluted lemon juice and patients in G1 received milk, immediately after the tracer injection, whereas patients in G2 received no intervention.

## Stress test protocol

A routine two-day protocol was used. Patients were stressed on day one and a rest study was done on day two. Patients were fasted for at least four hours prior to stress testing (usually overnight) and were required to abstain from caffeine-containing beverages and methylxanthine-containing medications for at least 24 hours. Caffeine and methylxanthines block the adenosine receptors on arterial smooth muscle cells, thereby limiting the effectiveness of vasodilator agents. Our department’s protocol is that we withhold caffeine in all patients, even if exercise stress is planned, in case there is a necessity to switch to pharmacological stress. Beta-blockers and calcium channel antagonists were withheld, where appropriate. The patients were haemodynamically and clinically stable for 48 hours prior to the test.

The stress modality (treadmill, dipyridamole or dobutamine) was chosen and implemented in accordance with the recent EANM guideline.^17^ Routine imaging for stress is carried out 30–45 minutes post tracer injection, however in our study some patients were imaged later due to the longer acquisition times with the addition of prone imaging, which is also a routine protocol in our department. All patients were imaged supine with their arms raised. Gated prone images were acquired after the gated supine stress images. The routine rest images were acquired 45–80 minutes post injection.

## Imaging protocol

SPECT imaging was performed using a double-head, rotating, large field-of-view gamma camera (GE Medical Systems Infinia hybrid system), equipped with a low-energy, highresolution collimator. SPECT images were acquired on a 64 × 64 matrix. Sixty images (25 seconds for rest, 20 seconds for stress) were obtained over a semi-circular 180° arc. Filtered backprojection was performed with a low-resolution Butterworth filter and no attenuation or scatter correction was applied. Transaxial tomograms were reconstructed and the images were re-orientated into three sets of orthogonal slices, including short axis, horizontal long axis and vertical long axis for each study.

## Data analysis

Two experienced nuclear medicine physicians (total experience 30 years) evaluated the raw data of the anterior (Ant) and left lateral (LLAT) views of both the stress and rest studies for the presence or absence of interfering infra-cardiac activity. Slice numbers 15 and 45 of the planar display from the SPECT acquisition were used in all patients to increase reproducibility. Slice 15 was chosen because of the best visualisation of the inferior wall of the left ventricle in the anterior projection, and likewise, slice 45 displayed the best projection for the inferior wall of the left ventricle in the lateral view. Observers evaluated the images simultaneously and were blinded to the clinical information as well as the protocol details. If there was a disagreement with the values obtained, a consensus was reached.

**Figure 1.**
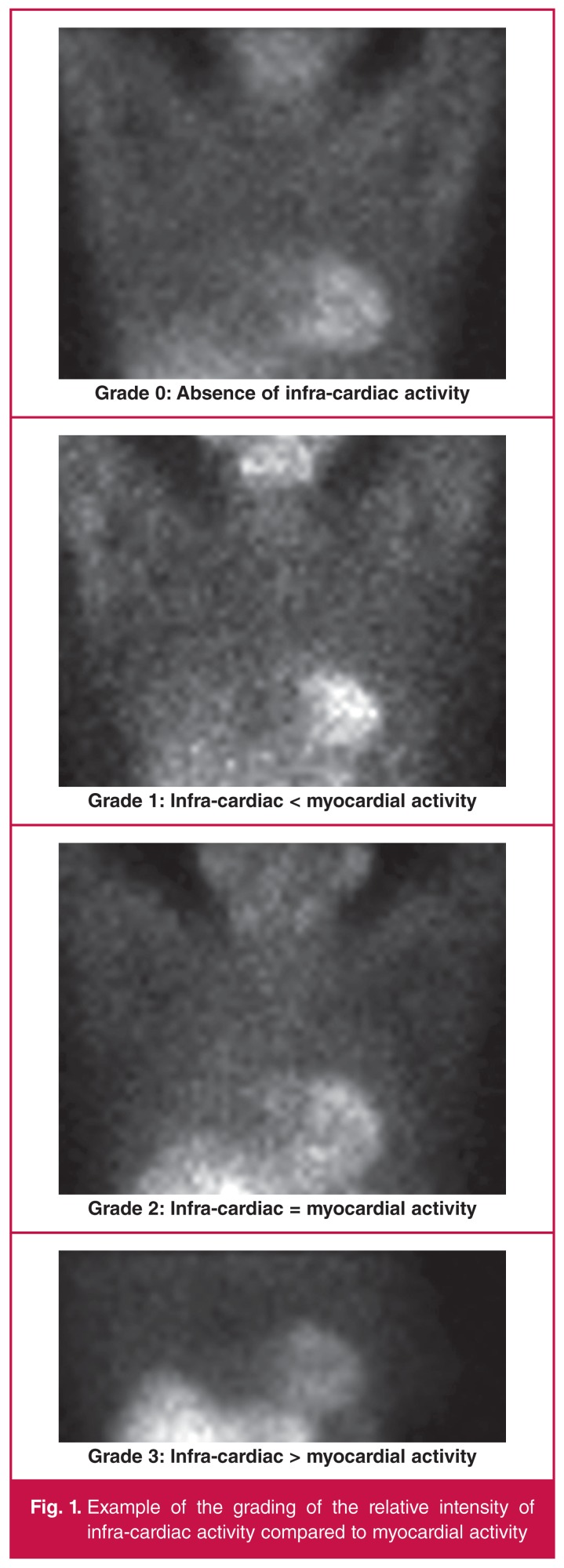
Example of the grading of the relative intensity of infra-cardiac activity compared to myocardial activity

The observers used visual and semi-quantitative assessment of the raw data of both stress and rest images, as previously used by Hofman *et al*.^8^ Visually, any presence of infra-cardiac activity was graded as ‘yes’ and the absence of infra-cardiac activity was graded as ‘no’. If the infra-cardiac activity was equal to lung background, it was described as absent. If infra-cardiac activity was present, it was graded as follows: 0: absence of infra-cardiac activity; 1: infra-cardiac activity less than myocardial activity; 2: infra-cardiac activity equal to myocardial activity; 3: infracardiac activity greater than myocardial activity (Fig. 1).

**Figure 2.**
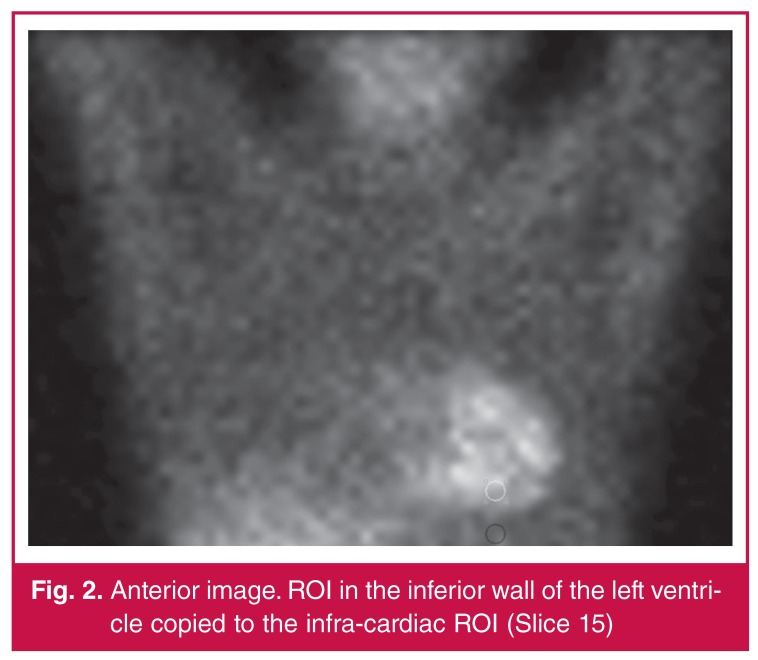
Anterior image. ROI in the inferior wall of the left ventricle copied to the infra-cardiac ROI (Slice 15)

**Figure 3.**
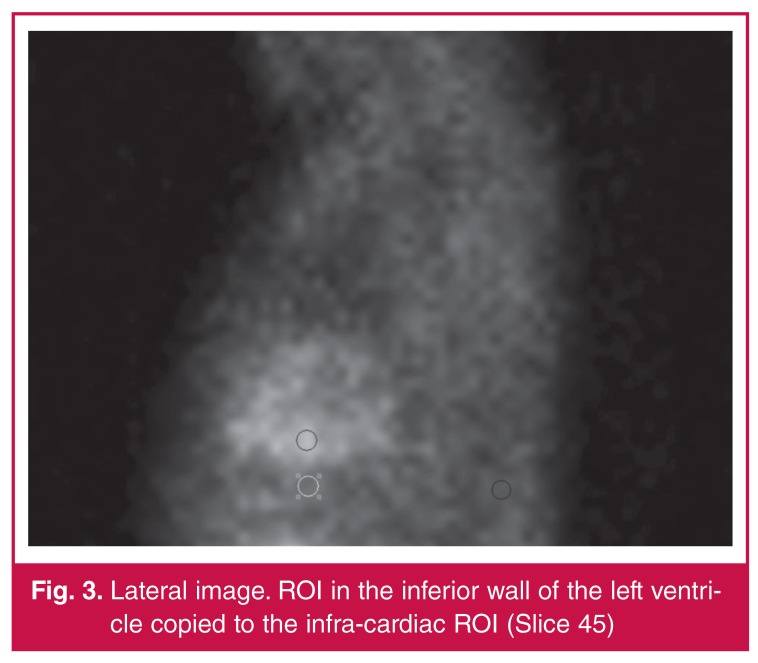
Lateral image. ROI in the inferior wall of the left ventricle copied to the infra-cardiac ROI (Slice 45)

For the semi-quantitative assessment, the total counts for the region of interest (ROI) were obtained on an anterior static image (slice 15). This ROI was manually drawn (six pixels wide). The same ROI was copied and pasted to the infra-cardiac area below the inferior wall of the left ventricle. On the same raw data, the images were rotated to a lateral view (slice 45), and the ROI was copied and pasted to the inferior wall and the corresponding infra-cardiac area (Fig. 2, Fig. 3). Regions of interest were copied between stress and rest studies of individual patients to increase reproducibility.

## Statistical analysis

Data were analysed using a Statistica 10 package (statsoft Inc, Tilsa, Oklahoma, USA).^18^ Descriptive results were presented as medians and range (normal or not normally distributed) for continuous variables. Categorical variables were summarised as frequencies and percentages. To assess the differences between continuous variables (age, counts in the left ventricle and infracardiac region at rest and stress) (not normally distributed), a Kruskal–Wallis test was used between the three groups, followed by the Bonferroni correction for two-by-two comparisons. *Post hoc* comparison of the mean ranks of all groups was performed. To compare frequencies of different categorical variables among the three groups, the chi-squared test or Fisher’s exact test were used when appropriate. Statistical significance was set at *p* < 0.05, and after Bonferroni correction at *p* < 0.016 for two-by-two comparisons.

## Results

Six hundred and thirty patients were randomised to receive either milk or lemon juice, or no intervention and their characteristics are shown in Table 2. Three hundred and thirteen patients received milk, 246 patients received lemon juice and there was no intervention in 71 patients. There were 304 females (48%) and 326 males (52%). The method of stress was exercise in 51% and pharmacological stress in 49% of patients. There was a statistically significant difference in the gender (*p* = 0.003) and ethnicity (*p* = 0.0002) indexes among the different study groups.

**Table 2:** Patient characteristics

*Characteristics*	*Total*	Total p-value*	*Lemon juice group (G0)*	*Milk group (G1)*	Control group (G2)**
Number	630		246 (39)	313 (50)	71 (11)
Mean age ± SD (year)			58.21 ± 11.42	62.03 ± 11.43	61.37 ± 9.02
Gender, frequency (%)		0.003			
Male	326 (52)		109 (44)	171 (55)	46 (65)
Female	304 (48)		137 (56)	142 (45)	25 (35)
Stress, frequency (%)		0.83			
Exercise	319 (51)		127 (52)	144 (46)	48 (68)
Pharmacological	311 (49)		119 (48)	169 (54)	23 (32)
Ethnicity, frequency (%)		0.0002			
Black	193 (30)		71 (29)	81 (26)	41 (58)
Caucasian	238 (37)		95 (39)	131 (42)	12 (17)
Indian	140 (22)		59 (24)	68 (22)	13 (18)
Coloured	59 (11)		21 (8)	33 (10)	5 (7)

*Total *p*-value represents the p-value for the three study groups.

In all three groups, infra-cardiac activity was present in the majority of patients, both on the stress and rest studies (Table 3). At stress, infra-cardiac activity was seen in 84.1, 84.5 and 97% of patients in G0, G1 and G2, respectively. At rest, infra-cardiac activity was seen in 91.7, 90.1 and 100% of patients in G0, G1 and G2, respectively. The visual assessment for the presence or absence of infra-cardiac activity showed a statistically significant difference among the three groups, both in post stress (*p* = 0.005) and at rest (*p* = 0.0063) (Table 3).

**Table 3:** Evaluation of infra-cardiac activity by visual assessment

*MPI*	*Total*	*Total p-value*	*Lemon juice group (G0)*	*Milk group (G1)*	*Control group (G2)*
Stress, frequency (%): presence of infra-cardiac activity		0.005			
Yes	528		201 (84.1)	257 (84.5)	70 (97))
No	86		38 (15.9)	47 (15.5)	1 (3)
Rest, frequency (%): presence of infra-cardiac activity		0.0063			
Yes	564		219 (91.7)	274 (90.1)	71 (100)
No	50		20 ( 8.3)	30 (9.9)	0

Presence of infra-cardiac activity was graded as ‘yes’ and absence as ‘no’ *Total *p*-value represents the p-value for the three study groups.

**Figure 4.**
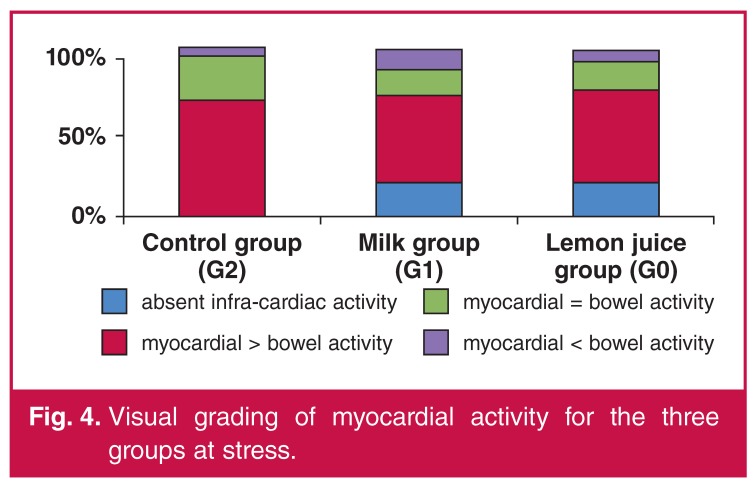
Visual grading of myocardial activity for the three groups at stress.

**Figure 5.**
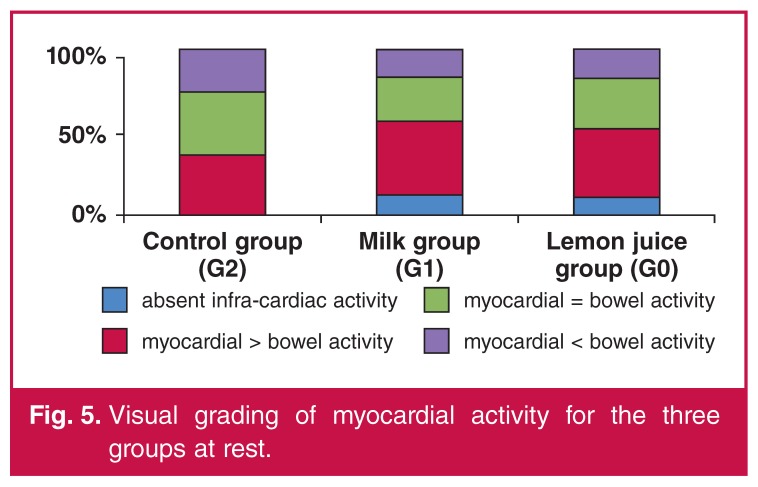
Visual grading of myocardial activity for the three groups at rest.

With regard to the quantitative grading, the majority of the patients had myocardial activity greater than infra-cardiac activity (at stress 74, 71 and 67% of patients for G0, G1 and G2, respectively) (Table 4). This finding was more evident in the groups with intervention, especially for the studies done at rest, and was more overt in G0 compared to G2 (*p* = 0.013). For the rest group, the majority of patients in G0 and G1 had less or equal interfering infra-cardiac activity. At rest, 81, 83 and 73% of patients for G0, G1 and G2, respectively, had myocardial activity greater than or equal to bowel activity. It was interesting to note that just over one-quarter of patients in G2 (27%) had infra-cardiac activity greater than myocardial activity, compared to G0 (19%) and G1 (18%) (Fig. 4, Fig. 5).

**Table 4:** Visual grading of the intensity of infra-cardiac activity versus myocardial activity

	*Grading*				
*MPI*	*0*	*1*	*2*	*3*	*Total p-value*
Stress, frequency (%)					0.0002
Lemon juice (G0)	38 (16)	138 (58)	46 (19)	17 (7)	
Milk (G1)	47 (16)	166 (55)	49 (16)	42 (14)	
Control (G2)	1 (1)	47 (66)	19 (28)	4 (6)	
Rest, frequency (%)				0.004
Lemon juice (G0)	20 (8)	100 (42)	73 (31)	46 (19)	
Milk (G1)	29 (10)	137 (45)	84 (28)	54 (18)	
Control (G2)	0	24 (34)	28 (39)	19 (27)	

*Total *p*-value represents the p-value for the three study groups. 0: absent infra-cardiac activity 1: bowel activity < myocardial activity 2: bowel activity = myocardial activity 3: bowel activity > myocardial activity

The difference in visual grading was also statistically highly significant for the three groups in post stress (*p* = 0.0002), and a similar difference was noted at rest (*p* = 0.004) (Table 4). The analysis of the quantitative assessment of the total counts for all subjects and their comparisons within and between groups are shown in Table 5. The median was obtained in each group for the variable, and their minimum and maximum values are included.

**Table 5:** Total counts in the myocardium and infra-cardiac area in the anterior and lateral view

*Region [median of total counts of ROI (range)]*	*Lemon juice group (G0)*	*Milk group (G1)*	*Control group (G2)*	*Overall p-value*
Stress, inferior myocardium anterior	634.5 (185–2648)*	733 (160–3260)#	553 (144–1566)	< 0.0001
Stress, infra-cardiac anterior	391 (79–1728)	429 (101–2551)#	364 (73–1308)	0.0106
Stress, inferior myocardium lateral	584 (103–2100)* (	673 (175–3913)#	534 (172–1693)	0.019
Stress, infra-cardiac lateral	419 (63–2119)	452 (90–2347)	393 (88–1805)	0.1129
Rest, inferior myocardium anterior	633.5 (186–8181)*	694 (36–2308)	586 (159–7171)	0.0089
Rest, infra-cardiac anterior	464 (83–2101)	512 (89–2329)	443 (145–1288)	0.088
Rest, inferior myocardium lateral	617.5 (109–2986)*	691 (32–2628)	612 (212–1897)	0.007
Rest, infra-cardiac lateral	488.5 (978–2672)*	552 (15–3037)	547 (126–1646)	0.020

*p < 0.05 between the lemon juice group (G0) and the milk group (G1). #p < 0.05 between the milk group (G1) and the control group (G2).

## Discussion

The use of ^99m^Tc sestamibi for MPI often results in increased splanchnic activity, which creates a major problem in the visual and quantitative interpretation of the inferior and inferoseptal walls of the left ventricle. Infra-cardiac activity arises predominantly from the liver, hepatobiliary system, bowel and/or gastro-duodenal reflux and can result in either an apparent increase or decrease in radiotracer uptake in the myocardium, especially in the inferior and infero-septal walls after reconstruction.^7^

Previous studies have been carried out, using both sestamibi and tetrofosmin MPI, with various agents used to reduce infracardiac activity, including the oral administration of various fluids or solid meals, and the use of pharmacological agents.^8^-^16^ The proposed mechanism of action is to fill the stomach, increasing the distance between the left ventricle and interfering infra-cardiac activity, or to increase liver clearance of radiotracer via gallbladder contraction.

In our study, the rationale for using lemon juice was that physiologically, acid-rich food or drink has the potential to facilitate hepatobiliary clearance of the bile by increasing the secretion of secretin, as was demonstrated by Peace *et al*.^12^ Milk was administered in the other group, as it was demonstrated by Hofman *et al*. that milk resulted in a significant decrease in the intensity of infra-cardiac activity.^8^ The mechanism of action is thought to be that administration of a fatty meal delays gastric emptying, resulting in increased volume in the stomach, and also that milk stimulates gallbladder contraction, resulting in movement of tracer from the liver to the duodenum.^8^ The reason for using milk and lemon juice in our study was the ease of availability, as well as the simplicity with regard to performing the study.

Our study, to our knowledge with the largest number of patients, compared whether the administration of full-fat milk or diluted lemon juice would improve the activity in the infra-cardiac region, and these interventions were compared to a group with no intervention. Our findings are overall in accordance with earlier studies in showing a decrease in infracardiac activity.^7^,^8^,^11^-^13^,^15^-^17^ The administration of milk or lemon juice showed a decrease in the presence of infra-cardiac activity, both for studies done at stress and at rest. Also it was noted that the image quality in the groups that had received an intervention (milk or lemon juice) was better in a greater percentage of patients, with images showing absent or infra-cardiac activity less than bowel activity (50% in G0, 55% in G1 vs 34% in G2).

It is known that infra-cardiac activity is more common in rest myocardial perfusion images,^5^ as was shown in our study, therefore our current protocol for rest MPI includes administration of 250 ml full-fat milk immediately after injection of the radiotracer. It is noted that when comparing patient satisfaction with regard to the interventions given, there was a general preference to the taste of milk compared to lemon juice.

Michael *et al*.^8^ compared milk versus water in reducing infracardiac activity in ^99m^Tc sestamibi MPI. He randomised 198 patients into two groups. One group had 150 ml chilled water and the other group had 150 ml milk five minutes after completion of the stress, and again five minutes before image acquisition. Patients also received 150 ml chilled water or milk five minutes after the rest injection, and again five minutes prior to image acquisition (total 600 ml of fluids for stress and rest images). There was a significant decrease in the intensity of infra-cardiac activity with milk compared to water. However the reduction in the intensity of infra-cardiac activity in the milk or water group did not translate into a statistically significant benefit in the image quality (*p* = 0.563 at stress and *p* = 0.502 at rest).

## Study limitations

By excluding almost a third of the recruited patients (274) from the original number (904), the powers in each group were not the same. There are numerous studies that have looked at interventions carried out together with the time of imaging to reduce interfering infra-cardiac activity. We did not look at the effect of time in addition to the interventions carried out. Also, even though the acquisition of prone images was part of our protocol, we did not analyse the raw data, therefore in the absence of prone analyses, the overall effect on a stress study with regard to the interventions carried out is not known.

## Conclusion

When using ^99m^Tc sestamibi, the infra-cardiac activity causes significant artifacts after reconstruction and may lead to errors in visual and quantitative assessment of myocardial perfusion, especially in the inferior and infero-septal walls. Our study demonstrated that the administration of milk or lemon juice resulted in a significant decrease in the intensity of infra-cardiac activity compared to a control group. There was a trend towards greater improvement in the group that received milk for the resting part of the study compared to the group that received lemon juice. However, we are not sure of the impact of the amount of either milk or lemon juice, as well as the timing of their administration on the study outcomes. Therefore, further studies are needed to determine the ideal amount and timing of administration of these interventions. Also, further studies should be done with regard to prone acquisition with these interventions.
